# The Health Narratives Research Group (HeNReG): A self-direction process offered to help decrease burnout in public health nurse practitioners

**DOI:** 10.3934/publichealth.2024009

**Published:** 2024-02-06

**Authors:** Carol Nash

**Affiliations:** History of Medicine Program, Department of Psychiatry, Temerty Faculty of Medicine, University of Toronto, Toronto, ON M5S 1A1

**Keywords:** nursing, sex roles, physician, profession, burnout, stress, COVID-19, narratives, self-direction

## Abstract

Founded in accordance with 19^th^ century sex roles and public health concerns, nursing evolved as other-directed, dependent on physician-focused diagnosis, prescription decisions, and public health advancements. The result of this other direction is that public health nurse practitioners have endured significant workplace stress resulting in burnout, especially during COVID-19. To help decrease their burnout, nurses require development of self-direction. The Health Narratives Research Group (HeNReG) has the potential to reduce burnout in nurse practitioners by encouraging the development of self-direction. The HeNReG process is presented through historically analyzed documents regarding reducing burnout in health researchers by developing self-direction including: (1) three years of archived year-end feedback results provided by participants, (2) archived participant responses to specific HeNReG-related writing prompts, and (3) a comparison of HeNReG results with the outcomes of resilience programs. The conclusion—the HeNReG offers an effective option for reducing burnout in health researchers that has the potential to decrease nurse practitioner burnout in a way that resilience programs do not. Tailoring the HeNReG process to public health nurses is discussed, inviting future research for reducing burnout in public health nurses.

## 1. Introduction

Nursing is a functionally autonomous, practical profession devoted to best practices in service. It demands integrity, continuing education, critical reflection, professional contribution, and interaction with other professionals [1],[2]. Unique to the profession of nursing is its foundation on 19^th^ century sex differences between women and men as well as the then popular and decidedly urgent concern with public health promotion and population disease prevention [3]—a concern that in the 21^st^ century arose again as a result of the 2020–2023 COVID-19 pandemic [4]. The nursing profession originated with Florence Nightingale's 1858 publication [5],[6]. However, Nightingale's ability to capture the public's imagination regarding the tenets of nursing was attributable to the accepted sex differences and the social changes that were then paramount regarding improving public health [7]. The role for women was epitomized as caring for others [8]. With the rise of professionalism in the mid-19^th^ century [9], the proper and ideal role of caring for others that women as a sex were seen to demonstrate arose concomitantly with the professionalizing of nursing [10]. The dichotomy between nursing as an autonomous profession and its dependence on entrenched sex roles has positioned nurses—especially those nurses overburdened as a result of public health demands related to COVID-19 [11]—to be a profession directed by others with the type of stress that leads to burnout [12]. This resulting burnout developed during the period when the role of physicians increasingly evolved to promote their power to diagnose and prescribe as the profession's primary purpose [13],[14]

With the recent evolution of sex to gender in social consciousness [15], the ideal representation of nursing in conjunction with women as a sex is no longer considered relevant to the profession [16] and is now viewed as counterproductive [17]. Although this change has had the positive result of an increase in those who identify as men [18] or as LGBTQ [19],[20] joining the nursing profession, it has also questioned the value of the original identifying sex feature of nursing as representing the ideal woman [21] and imposed a different other-directed ideology external to their profession. Also apparent with the migration of sex to gender, the most valued quality of medical students is now their empathy [22],[23], in contrast to their power to diagnose and prescribe. Consequently, the clear distinction between the profession of medicine and that of nursing upheld throughout the 20^th^ century has now become blurred [24], with nurses taking on more roles that previously were confined to physicians [25], especially regarding public health requirements during the COVID-19 pandemic [26], representing another impediment to the self-direction of nurses. Furthermore, physicians in training are attending to a greater number of activities that have in the past only been considered within the realm of nurses [27]. The obvious divisions of nursing as a profession for women and medicine as a profession beyond care for men have been challenged and continue to be dissolved [28]. Although these changes bring additional opportunities for greater diversity in its practitioners [29],[30] and greater collaboration between nursing and medicine [31], they have also served to increase the stress associated with the nursing profession [32] by placing more other-directed burdens on them.

Since Nightingale's 1858 publication, the nursing profession has proclaimed the terms of what makes it unique [33] to the extent that, between 1858 and 1910, when nursing was seen to epitomize care and the ideal of being a woman—and stood above medicine in the clarity of its role [34]—nurses were lauded for their well-defined role [35]. Socially and professionally, changes have undermined this respect for nursing [36]. Thus, the most effective protection for nurses to reduce their stress is one that reestablishes clarity in being a nurse by developing public health nurse practitioners' self-direction, leading to burnout reduction [37],[38]—something that goes beyond the ways to enhance resilience [39],[40] that have been increasingly promoted [41]–[43] with respect to burnout in nurses.

The World Health Organization considers burnout a syndrome in the occupational context resulting from unsuccessfully managed chronic workplace stress characterized by feelings of energy depletion or exhaustion, increased mental distance or feelings of negativism or cynicism related to work, and reduced professional efficacy [44]. COVID-19 has been a significant factor in the burnout experienced by nurses [45] since March 11, 2020, when the pandemic began [46]. The effect of COVID-19 has been the public recognition that what is most important to improving public health is improving the mental health of public health providers diminished as a result of burnout [47]. Burnout in public health nurse practitioners was especially prevalent during the early months of the COVID-19 pandemic when the main risk factors were younger age, decreased social support, low family and colleagues readiness to cope with the COVID-19 outbreak, increased perceived threat of COVID-19, longer working time in quarantine areas, working in a high-risk environment, working in hospitals with inadequate and insufficient material and human resources, increased workload, and lower level of specialized training regarding COVID-19 [48].

Although they are related, burnout is not equivalent to a lack of resilience. How burnout differs from lacking resilience is that resilience—as a trait, a process, or an outcome—is independent from work. As a Big Five personality trait, resilience is fixed and stable over time and, therefore, cannot be modified [49]. Whereas, when viewed as a process, resilience can vary across context and time [50]. Considered as an outcome, it is the result of successful coping [51]. Regarding public health nurse practitioners, this means they may lack the trait of resilience but not feel burned out [52]. It also means that attending to improving their resilience if they are trait deficient is unlikely to be effective. If viewed as a process, the resilience of these nurse practitioner may improve with training in relation to some aspects of their life but possibly not in relation to their burnout [53]. Lastly, if public health nurse practitioners have an outcome of resilience, they may be coping successfully but still feel mentally distant, negative, or cynical in connection to their work [54]. Currently, there is no certainty regarding the nature of the trait or skill of resilience [50]. For these reasons, a focus on resilience training to reduce burnout is likely insufficient and may be entirely ineffective for those who are trait deficient.

Self-direction is a concept that arose in the field of adult education [55] during the 1970s [56] and is still a term widely used in the field today [57]. Common to the various definitions of self-direction is the importance of having a level of freedom without control from others [58]. Regarding the original sex division of nurses, self-direction is not traditionally part of women's role; therefore women (and nurses as epitomizing the role of women) do not usually see themselves as agents of self-direction in their lives [59]. In focusing on moving beyond past limitations from their professional dependence on entrenched sex roles, self-direction regarding public health nurse practitioners relates to enhancing these nurses' energy, commitment, and organizational ability to successfully take responsibility for their work [60]. In this way, self-direction for such nurse practitioners translates into engaging with work in such a way that perceived challenges do not negatively affect public health nurse practitioners' relationship to their work [61].

Assessing clinician burnout to determine effective interventions has become a topic of great interest as a significant number of advanced nurse practitioners (59%) have experienced burnout—yet, there are few studies specific to them [62]. In this regard, the research question of this study is: In what way might a group narrative development process support public health nurse practitioners in developing self-direction to help reduce their burnout in contrast to resilience programs? The purpose of this study is to present an offering that has the potential to lessen burnout in public health nurse practitioners—the Health Narratives Research Group (HeNReG)—by developing self-direction in group participants. This method, developed by the author, was originally created to help reduce burnout in health researchers self-identifying as experiencing burnout. Based on a historical analysis of the archived results of the three years it was an in-person group, it is hypothesized that the process employed for the in-person group is one that might be successful in reducing burnout in nurse practitioners, especially those who have been over-burdened as public health practitioners during COVID-19. The archived results of the HeNReG will be analyzed historically and include both the themes provided from the year-end feedback forms of the participants, as well as group members' responses to HeNReG-specific writing-prompts. Following this analysis, a comparison will be made of the suitability of the HeNReG in increasing self-direction and reducing burnout in comparison with programs designed to improve resilience. Finally, recommendations are offered regarding the application of the process to public health nurse practitioners, pointing to future research in this area.

## 2. Materials and methods

All features of the HeNReG that are important to its creation and continuation will be analyzed with a historical research method using primary sources previously compiled and archived by the author. Historical research methods have been defined as “the class of techniques used for the compilation, description, and critical analysis of primary and secondary historical sources with the intention to provide a contextualized explanation and interpretation of the phenomenon of interest” [63]. In following a historical research method, the headings of this section are particular to the HeNReG and are not structured as would be expected of an empirical research study [64]. The methods and materials instead follow the logical progression of the historical development of HeNReG aspects.

### 2.1. Description of the HeNReG

The HeNReG is offered here as a potentially useful process in reducing the burnout experienced by nurse practitioners by developing their self-direction. It was a process developed by this author through the Department of Psychiatry at the University of Toronto as part of the Health, Arts and Humanities Program [65] as a voluntary, non-credit group meeting held throughout the academic year, open to any member of the university community interested in health research, self-identifying as experiencing burnout. As an in-person group with a particular, refined structure, it was offered from 2016–2019. The results concerning this group have been published elsewhere [66]–[70]. For those interested in offering this type of group, it is important to note that diversity of membership is sought and encouraged [71]. This type of group epitomizes support of the 4Cs considered 21^st^ century skills [72]: critical thought, communication, cooperation, and creativity [73]. It represents a weekly, two-hour opportunity for university researchers, ranging from undergraduates to full professors, to take the personally relevant stories that initiated their commitment to health research and develop them into narratives with a particular point of view through responding to writing prompts. The process includes both participant self-reflection and the willingness to share one's story as well as gain additional insights from the rest of the group. Although the experience level may differ among the researchers, members are asked to treat each other as equals in the role of group participants as this has been found to enhance the collaborative process [74] and is necessary for developing self-direction [75].

#### 2.1.1. Ethics approval of research

As historical research of what represent yearly qualitative focus groups, involving participant observations of University of Toronto students, faculty, staff, and alumni, ethics approval was not required for research regarding the HeNReG as per the University of Toronto Guideline [76].

### 2.2. Historical account of group participation

Upon seeing the yearly notice for this offering posted online through the University of Toronto Health, Arts, and Humanities Program [65] or meeting the facilitator personally, participants indicated their interest in joining the group by emailing the facilitator (the author of this article and founder/facilitator of the HeNReG) along with their reason for wanting to join the group. If continuing their interest after a request to join, the facilitator sent the interested person a yearly-updated document (see Supplementary) outlining the operation of the group. The document also specified that by agreeing to join, participants acknowledge that the facilitator may use the archived material of the group anonymously for research presentations or publications. If agreeing to the conditions of the document, the interested person sent an email in response to the facilitator indicating that they would like to join the HeNReG. Their informed consent to join the group is written in this manner. Participants may attend as many or as few sessions as they prefer. The group is facilitated in a manner similar to that assumed by a health and wellness coach [77].

Over its three years of in-person operation, between 12 and 22 researchers participated in the HeNReG pre-COVID-19 in any one academic year. During this period, the group met in the occupational therapy meeting room at the Toronto Mount Sinai Hospital, one of the University of Toronto teaching hospitals. The disciplines represented by the participating researchers during these three years are presented in Table 1, including the total number of participants for each year. This information is presented as an example of the types of disciplines that have the potential to be interested in participating in the HeNReG. As the historical method being used considers group membership a material for the method, this list is presented as archived historical material, not as results. The results, to follow in the next section, will concern only the responses of the participants in the form of feedback and in relation to the responses to the writing prompts.

**Table 1.** Discipline represented by each participant of the HeNReG for the years when the common group process was completed in-person at the Toronto Mount Sinai Hospital, from 2016–2019, plus the total participants.

**Discipline**	**2016/2017**	**2017/2018**	**2018/2019**
Adolescent medicine		1	
Bioethics	1		
Bioinformatics			1
Chemical engineering		1	
Community health		1	
Comparative literature	1		
Computer science			1
Creative writing	1	2	
Critical theory		1	
Disability studies			1
Diaspora studies		1	1
Economics		1	1
Education		1	1
English		1	1
Engineering			1
Exercise health	1		
Health and safety		1	1
History of medicine	2	1	1
Marketing	1		
Medicine		2	
Medical information		1	1
Narrative research		1	1
Neuroscience		2	2
Nursing			1
Palliative care		1	
Pharmacy	1		
Psychology	1		
Psychotherapy		1	1
Socially engaged art	1	1	1
Social work	2		
Student services		1	
**Total participants**	**12**	**22**	**17**

Undergraduate students, graduate students, researchers (non-faculty), and faculty were represented in every one of the three years. The breakdown of these academic levels for each year that the HeNReG was completed in-person (pre-COVID-19) is represented in Table 2, including the total and percentage of each level and the total number of participants. Again, this information is presented to demonstrate the range and proportion of academic levels that have the potential to show interest in participating in a HeNReG as material for the historical analysis of this group. It is not presented as results. In contrast, the results of this study will concern only the answers that participants provided to the feedback forms and the responses to the writing prompts.

**Table 2.** Academic level of each participant in the HeNReG for the years when the common group process was completed in-person at the Toronto Mount Sinai Hospital, from 2016–2019, plus the total participants and their percentage.

**Academic level**	**2016/2017**	**2017/2018**	**2018/2019**
Undergraduate student	4 (33.33%)	7 (31.82%)	6 (35.29%)
Graduate student	1 (8.33%)	1 (4.54%)	3 (17.65%)
Researcher (Non-faculty)	5 (41.67%)	12 (54.55%)	7 (41.18%)
Faculty member	2 (16.67%)	2 (9.09%)	1 (5.88%)
**Total participants**	**12**	**22**	**17**

What can be noted from both the range of disciplines, and the academic levels represented in the three years of the HeNReG in-person meetings, is that this group was diverse each year in these respects. As the group was specified for health researchers, it is relevant that the greatest percentage of those who participated over the three years represented non-faculty researchers. This level included assistant researchers, associate researchers, and visiting researchers to the university. In every year, the members included representatives from the humanities, social sciences, physical sciences and life sciences—from those just starting out in their careers to faculty members. What was common among these participants was their interest in health research and their self-identified feeling of burnout in relation to their health research. Those reading this historical account interested in creating such a collaborated self-directed group at their institutions should note that the focus on diversity in both disciplines and represented and in levels of expertise is considered imperative to the intention and success of the group in helping participants decrease their research burnout [78].

### 2.3. Philosophy of the group

The philosophy of the group is unique, and depends on a particular interpretation of truth introduced by the facilitator. With the foundational philosophy for the group (reflected with recent empirical research [79]), there are two avenues to truth through research. These may be analogized into two methods of approaching a landscape with barriers. One avenue is disciplinary [80] and sees obstacles in the landscape as barriers to eliminate by climbing higher. In disciplinary research, higher views supersede lower ones as disciplinary research is necessarily hierarchical. In this analogy, the purpose of discipline-based research is to create the most accurate aerial view of the landscape by overcoming and rising above the obstacles in the landscape. In contrast, narrative research [81] is the term used to describe the second method of traversing the landscape of truth. Regarding the HeNReG, this type of research views obstacles in the landscape as landmarks to use in developing routes around these features where each person's point of view is considered equal, as all perspectives are seen as necessary to view the landscape in its entirety. As a narrative research group based on a non-hierarchical structure, the group is also non-competitive [66] as collaborative groups are found to be the most effective and advantageous [82] in this regard. The routes created from one point of view to another are added together to complete the landscape map. The collaborative purpose of narrative research in the HeNReG is to create as many routes as possible from one point of view to another, a reason why diversity is encouraged in group membership. For those unclear of how this might work if starting such a group, this process can be visualized as similar to how a “street view” is constructed in something like Google Maps [83].

### 2.4. Process followed

At the beginning of each session, a prompt—pre-designed by the facilitator—was provided, to which group members were to write spontaneously in response for five minutes, self-directing their interpretation of the prompt. The initial prompt provided at the first meeting of the academic year asked each person to describe themselves with respect to their research related to health. In the weeks to follow, the prompts were designed to ask group members to first consider what is most objective with respect to their research related to health as explained in their initially-shared self-descriptive story, then, as the weeks progressed, the prompts regarded questions that began as objective and increasingly became subjective. Although the individual prompt questions changed each year, the order of the type of questions asked remained the same: when, where, who, what, how, why. More than one session was devoted to each type of question—four weeks for the more objective questions (when, where, who and what questions), additional weeks for those questions evoking more subjective answers (five for how, and six for why questions). This structure of question asking was intended by the facilitator to help participants—through a non-threatening and ever psychologically-deepening way—develop their stories into a larger narrative with a particular point of view that would help to sustain their research throughout their career. In using this method, the meetings supported the critical thought of the 4Cs [72],[73].

Once participants completed their written response to the weekly prompt, each person was asked to read their response, one-by-one. After a participant had read their response to the prompt, each other member was given the opportunity to provide a question to the person who had read their response to further clarify what was read. The questioning process worked its way around the circle of how participants happen to be seated. The only requirement of the question asked was that it must begin with the same word of the week, i.e., if a “who” question was asked, each person then asked a clarifying “who” question of the reader.

In a group constructed to follow this process of the HeNReG, devising these questions requires the creativity of the 4Cs [72],[73] on the part of the participants. If a group member does not have a question, that person may pass. If a group member cannot think of a question right away, after each of the other members one at a time has provided a question, then the person who passed is given an additional opportunity to pose a question. The purpose of this question-asking process is to permit those who ask the questions to see the landscape from the point of view of the reader. For the participant who is the focus as reader, answering the questions, the objective is to revisit their point of view to picture it in greater detail. In each exchange, the development of communication of the 4Cs [72],[73] is promoted.

Keeping with the non-competitive nature of the group, people participating in-person waited their turn to speak and did not put up their hands [66]. Members aimed to make their questions as short as possible so everyone had the chance to speak. After each question was asked, the person who had read their reply to the prompt answered the question asked of them. When each person had asked a question of the reader, the person who had read gave a summary of how these questions may have helped them clarify their research. The facilitator noted down all the questions that people asked, and their replies, after which it was the next person's turn to read. Depending on each member to wait their turn and listen carefully to others requires the cooperation of all—the final aspect of the 4Cs [72],[73].

Table 3 represents each of the various prompts that were provided to researchers regarding the more objective when, where, who, and what questions for the three years the HeNReG was offered using the above-mentioned process. Table 4 presents the various prompts that were provided to researchers regarding the more subjective how and why questions for the three years the HeNReG was offered using the above-mentioned process. These prompts are provided as examples of the types of prompts that facilitators of such groups can use to create their own collaborative self-directed groups similar to the HeNReG.

In each of the years of the in-person offerings of the HeNReG (pre-COVID-19, as once the pandemic began the group moved online), the facilitator developed the six “why” prompts that corresponded to asking why questions related to each of the six different types of questions that were asked during the academic year. As such, the first “why” prompt related to a “when” question. Those that followed concerned “where”, “who”, “what”, “how”, and “why”, in that order. The purpose in directing the “why” questions in this manner evident in Table 4 was to ensure that the participants were requested to consider a subjective aspect of all the objective and increasingly subjective types of prompts provided at the HeNReG. As well, in both of the last two years, the facilitator took the opportunity of posing prompts directly related to the way in which the HeNReG in particular might be valuable to the participants. Table 4 notes two “how” questions, and one “why” question in 2017/2018. By the final year of the in-person meetings, the facilitator developed these questions related to the HeNReG more systematically by asking one HeNReG-related question regarding each of the six different types of prompts. In this way, participants were prompted to self-reflect on the six different ways in which the HeNReG might be of value to them. This was done to reinforce with the participants the value of the method that was being presented to the group members in lessening their research-related burnout. It is suggested that those starting their own HeNReG-like groups similarly follow this aspect of the process to gain access to how their process is received by group participants.

**Table 3.** Yearly more objective prompts concerning when, where, who and what, created by the facilitator and provided to group members in the three years of the HeNReG's in-person meetings at the Toronto Mount Sinai Hospital, listed in the order in which they were posed during each academic year.

**Prompts provided by the facilitator in each of the three years of in-person HeNReG meetings**		
**2016/2017**	**2017/2018**	**2018/2019**
When did things start to change regarding your research related to health?	When did you first think your research related to health was important?	When did you feel you had the energy to accomplish what you wanted to do regarding your research related to health?
When did you want to give up on your research related to health?	When did you have a new idea for your research related to health?	When did you think you needed to relocate to continue your research related to health?
When did you first get the idea to do you research?	When did you decide you had to do something?	When did you think the HeNReG could be of help to you?
When have you faced opposition in your research related to health?	When should you stop your research related to health?	When did you clarify your goals for your research related to health?
Where in the body is your research related to health located?	Where do you feel most satisfied discussing your research related to health?	Where were you when you first thought you needed the HeNReG to help with your research related to health?
Where will you go with your research related to health?	Where do you write about your research related to health?	Where have you felt inspired regarding your research related to health?
Where do you get your information?	Where have you misplaced something in your research?	Where did you meet with difficulties in pursuing your research related to health?
Where do you go when you are stressed?	Where will you be when you reach your goal regarding your research related to health?	Where did you reveal too much regarding your research related to health?
Who are you regarding your research related to health?	Who has been inspired by you in your research related to health?	Who has been behind your research related to health from the start?
Who benefits from your research related to health?	Who have you neglected in your research related to health?	Who have you told about the research practice used at the HeNReG?
Who plays a part in your research related to health?	Who are you most like in the way you conduct your research related to health?	Who was your role model for your research related to health?
Who have you encountered in your research related to health?	Who has created work you want to study for your research related to health?	Who gave you reason to question pursuing your research related to health?
What is unique about your research related to health?	What is your most cherished memory regarding your research related to health?	What were you intending when you started your research related to health?
What would success look like regarding your research related to health?	What should you be doing right now regarding your research related to health?	What do you most want answered that you've yet to find out?
What problem are you solving with your research related to health?	What unexpected thing happened regarding your research related to health?	What's the best way to get things done for your research related to health?
What do you value of your research related to health?	What do you identify with in your research related to health?	What would improve the usefulness of the HeNReG to your research related to health?

**Table 4.** Yearly more subjective prompts concerning how and why, created by the facilitator and provided to group members in the three years of the HeNReG's in-person meetings at the Toronto Mount Sinai Hospital, listed in the order in which they were posed during each academic year.

**Prompts provided by the facilitator in each of the three years of in-person HeNReG meetings**		
**2016/2017**	**2017/2018**	**2018/2019**
How do you experience time regarding your research related to health?	How did you feel when you first started your research related to health?	How do you know you are on the right track with respect to your research related to health?
How have you reduced the scope of your research?	How do you gather the resources you need for your research related to health?	How has encouraging doodling at the HeNReG meetings affected your research related to health?
How have you sustained your interest in your research?	How did you become aware of the HeNReG?	How do you focus on listening to others?
How have you overcome institutional barriers with respect to your research?	How could the HeNReG be of more help to you regarding your research related to health?	How do you follow through on completing you research related to health?
How many ideas do you have regarding your research?	How do you deal with criticism regarding your research?	How do you physically organize your research related to health?
Why did you start your research at the time you did?	Why did you feel you needed to change when you approach your research?	Why do you work on your research related to health when you do?
Why is the University of Toronto a place to further your research related to health?	Why do you continue researching in your current location?	Why is Toronto the best place to conduct your research related to health right now?
Why do you think you can conduct research related to health?	Why have you wanted more from participants in your research related to health?	Why is it important to be who you are as a researcher?
Why do you value your research related to health?	Why has the HeNReG contributed to you feeling anxious or overwhelmed?	Why are you having difficulty doing what you want as a researcher?
Why have you developed your particular methods in your research related to health?	Why do you search out different points of view for your research related to health?	Why does it matter how you conduct your research related to health?
Why have you retained your passion for your research related to health?	Why is it important to pay attention regarding your research related to health?	Why has the HeNReG helped you understand why you value your work?

### 2.5. Drawing and doodling as an additional aspect of the HeNReG

HeNReG participants spontaneously began drawing and doodling on their own during the meetings from 2016–2018. Based on this self-direction of participants in expressing themselves, a practice was begun in the 2018–2019 year of the in-person meetings of the facilitator specifically encouraging drawing and doodling during the course of the weekly meeting. This was done through the facilitator providing the group with artists materials and paper, and suggesting participants draw or doodle during the meeting, thus offering an additional outlet to express the creativity promoted by the 4Cs [72],[73] to help put members at ease [84]. The purpose of encouraging group members to draw or doodle was that it was noticed within the group to reduce the anxiety and/or depression of those who found this type of self-expression to be a novel experience in an academic setting, helping them to concentrate more deeply on questions to ask the current reader [84]. That this practice helped to reduce stress in the participants of the HeNReG has been noted elsewhere [85],[86] (see the first article cited here for examples of the doodles produced during the 2016–2019 years). Sometimes drawing prompts were provided alongside the writing prompts. These might have been provided by the facilitator, or any other member of the group, although they were usually provided by an artist-member of the group. Unlike the writing prompts, which were to be completed in five minutes, participants responded to drawing prompts over the entire two-hour meeting, or chose not respond to them at all. No one was required to draw. When there was no drawing prompt, members were encouraged to doodle on their own instead. At the end of the meeting, members were asked to describe their drawings or doodles one at a time going around the circle, although this was not required. The facilitator then noted down all the descriptions. Those wanting to replicate the doodling aspect of this this group are encouraged to use a similar method to this that has been presented here.

### 2.6. Record keeping and storage

Part of what was important to the in-person meetings of the HeNReG between 2016–2019 is the extent of records that were kept and how they were stored. To join the group, each participant was required to have a Facebook account and to “friend” the facilitator. The purpose was to create a yearly private Facebook group (at the time, called a “secret group” by Facebook [87]) to post the prompts, each person's response to the prompts, the questions they were asked, and those they asked of others. As well, any drawings and/or doodles were also posted along with the questions asked of these creations plus the replies given to the questions by their creators. To keep these records necessitated the facilitator taking detailed notes during the meeting and then transcribing them to the private Facebook group. Given that collaborative self-reflection is so important to the process of the HeNReG, for those interested in replicating this method, it is suggested that the person assuming the role of facilitator ensure that detailed records be kept of the group's proceedings and that they are stored in a place that is accessible to participants at any time during the process of the group, and once the process is complete—such as a private Facebook group.

In addition to the private Facebook group, the accompanying Messenger app permitted messages to be sent to participants. As a result of the ability of these two platforms to work in combination [88], members who were unable to be at the meeting in person any one week were able to participate in the weekly meeting by replying via Messenger to the writing prompt the facilitator gave to each member weekly through Messenger a day before the meeting. Their replies were then read by the facilitator at the meeting and questions to the physically absent member were noted by the facilitator who later posted them to the private Facebook group after the meeting, permitting the absent member to read and respond to them online. The private Facebook group gave members the ability to access the private Facebook group when they had time: after the meeting, sometime during the week before the next meeting, or any time after that. Members could scroll through the postings of the entire academic year using the private Facebook group. As such, there are instances when members found things posted that they wanted to respond to months after the posting was originally made, to which they could then reply.

Near the end of each academic year, the facilitator created a year-specific feedback form using Google forms based on a similar feedback form used by other offerings associated with Health, Arts and Humanities in the Department of Psychiatry of the University of Toronto Temerty Faculty of Medicine. During the last meeting of every year, the facilitator informed the group members that a link would be sent to them via Messenger of the feedback form for them to complete. Participants were asked to complete the form as soon as possible. If the completed feedback forms were not received within two weeks, reminders were sent weekly to the group member until the form was received. At the most, two reminders were necessary to receive all feedback form returns. As Google forms, these completed feedback forms are stored in perpetuity as part of the facilitator's Google forms account.

The in-person aspects of the meetings ended with the COVID-19 lockdown when all meetings were required to move online at the University of Toronto, March 13, 2020 [89]. The private Facebook group for the year continued. Nevertheless, the results that will be reported will be only for the pre-COVID-19 years, when the entire process was available in-person at weekly 2-hour meetings, as only these years are entirely comparable.

### 2.7. Flowchart of the creation and maintenance of a HeNReG

**Figure 1.**
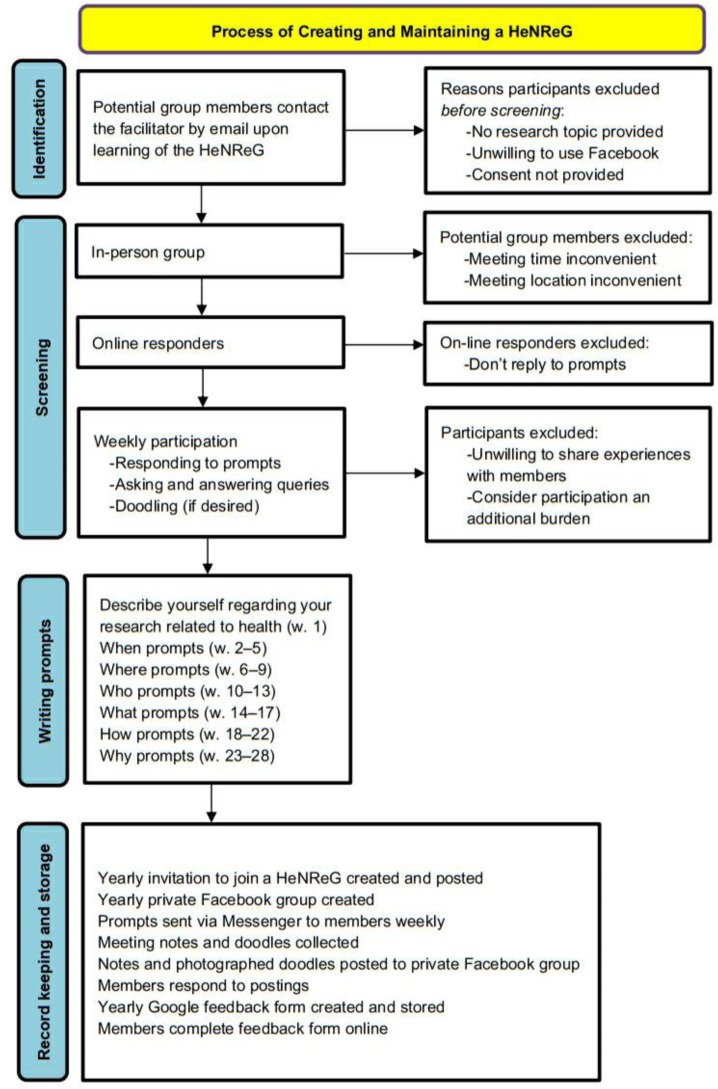
Flowchart of creation and maintenance of yearly HeNReG including identification and screening of members in relation to aspect of the group, writing prompts (order and number of weeks), and record keeping and storage (order and method).

Figure 1 presents the construction and maintenance of a HeNReG using the template recommended by the Preferred Reporting Items for Systematic reviews and Meta-Analyses (PRISMA) [90],[91]. Although this historical analysis of the HeNReG is not a systematic review, this flowchart template was selected because it has been standardized and is considered superior in this regard [92].

## 3. Results

The first results to be presented will be the narrative portion of responses that participants gave yearly to the feedback form distributed to group members on Messenger after the last HeNReG meeting of the academic year. Second, the responses to HeNReG-related prompts will be considered. The purpose of presenting these archived results is so those interested in starting a similar group can see the types of responses participants provided in considering various aspects of the group's process.

### 3.1. Narrative responses from feedback forms

The themes of the narrative responses that HeNReG participants provided on the year-end feedback forms in each of the three years between 2016–2019 have been tabulated regarding two questions: “How was the group valuable to you as a researcher?” (Table 5), and “How might the HeNReG be of help to you in the future?” (Table 6).

**Table 5.** Themes of narrative responses provided on the year-end feedback forms to “How was the group valuable to you as a researcher?” for the three academic years the HeNReG met in person at the Toronto Mount Sinai Hospital in order of receipt of members' feedback forms.

**Themes mentioned in feedback forms**	**2016/2017**	**2017/2018**	**2018/2019**
Learned about myself	1		
Helped me organize my thoughts	1		
Motivated me to push my research further	1		
Gave me a deeper connection to my research	1		
Provided the perspectives of other researchers	2	1	4
Allowed me to focus on the details of my research	1		
Learned how to overcome my research challenges with the help of others	1		
Better able to define my research direction	1		
Made me more introspective regarding my research	1		
Gave me perspective in how to approach my research	1		
Showed how I can improve as a writer and speaker	1	2	
Provided first-hand experiences	1		
Gave regular repetition of a useful method	1		
Developed skills in ways not available through workshops or seminars	1		
Provided an enjoyable experience		2	
Showed me how to incorporate difference experiences		1	
Helped provide me with ideas to explore		1	
Showed me the importance of asking “why?”		1	
Moved me from being unsure to knowing how to respond		1	
Clearly defined the truth I have been pursuing		1	
Created the perfect conditions to surge my development as a researcher		1	
Expanded my critical thinking skills		1	
Encouraged me to think ahead and outside the box		1	
Showed the value of a diverse group in gaining perspective		1	
Encouraged self-reflection on research			4
Helped in greater understanding of my research			2
Sharpened thinking about research			2
Invited a broader view of research			3
Gave a safe space to verbalize ideas about research			1
Challenged my thinking about research			1

**Table 6.** Themes of narrative responses provided on the year-end feedback forms to “How might the HeNReG be of help to you in the future?” for the three academic years the HeNReG met in person at the Toronto Mount Sinai Hospital in order of receipt of members' feedback forms.

**Themes mentioned in feedback forms**	**2016/2017**	**2017/2018**	**2018/2019**
Moving from the objective to subjective helped me organize my thoughts	1		
Improving my ability to make plans regarding my research	1		
Improving my research through the exchange of knowledge	1		
Offering different points of view	1		1
Polishing my soft skills	1		
Giving me more confidence in sharing my views	1		
Helping me question my habits	1		
Receiving objective perspectives and constructive suggestions in a safe community	1		
Being able to discuss and clarify perceptions	1		
Learning communication skills	1		
Learning the importance of views of people from different backgrounds to improving my research	1		
Getting great feedback	2		
Coming to know of others' research experiences	1		
Encouraging me to take personal responsibility	1		
Making me a better writer and speaker	1		
Showing me it is possible for diverse groups to meet and function well in sharing their views		1	
Providing a unique philosophy		1	
Giving me a new way to explore self-reflection		1	
Providing a holistic understanding of reflecting on research		1	
Providing me with a community of researchers		1	
Learning to read my thoughts out loud		1	
Becoming comfortable as a researcher		1	
Being more structured in what I write in thinking that someone will be reading it		1	
Assisting me in enhancing my research techniques		1	
Being able to interact with group members in the future		1	
Strengthening the quality of my research		1	
Showing a method for continuous self-growth		1	
Being there for me when I needed to overcome a research challenge		1	
Learning the importance of sharing my work		1	
Opening up discussion			1
Meeting interesting participants			1
Using the structure of weekly prompts to guide my self-reflection			1
Decreasing my confusion as I decide what should be my focus in my research			1
Sharing resources			1
Making me more open-minded			1
Permitting me to grow as a researcher			1
Listening to others and giving feedback			1
Keeping me updated on interesting topics in various fields			1
Encouraging more collaborative artistic creation in my research			1
Understanding and respecting different points of view			1
Continuing with creative reflection			1
Helping me plan my research			1

Consolidating the categories of the information provided in the themes noted in Table 5 and Table 6 produces three results: self-development as a researcher, the collaborative process, and the process of the HeNReG. These are noted in Table 7.

Examining Table 7, it can be identified that the larger the number of group members in any one year: (1) the fewer the individual comments made by participants, (2) the larger the number of participants who focused on the HeNReG regarding the process, (3) the fewer the number of participants who specified the importance of the collaborative process, and (4) the fewer the number of participants who noted their ability to develop as a researcher. What these results point to is that, with the larger group, the value of the process itself is more evident. In contrast, with the smaller the group, it is more likely that participants will focus on their own self-development as a researcher. On the other hand, for the value of the collaborative process to be the focus, the middle-sized group provides results that are most attuned to this aspect. Consequently, if setting up a group similar to the HeNReG, the size of the group sought will depend on the type of change that is most likely to result in self-direction. If the aspect to modify is seen to be self-reflection, the group should be smaller. If, instead, the concern is to develop collaboration among different colleagues, the middle-sized group would be most appropriate. Lastly, if the interest is in establishing an easy-to-use method as valuable for structuring thought, then the larger sized group might be most effective. It has been noted that a bundled package is necessary to reduce burnout—with colleague collaboration in particular and communication skills training as aspects of this bundle found to be helpful [93].

**Table 7.** Consolidated categories for Table 5 and Table 6, including the total number of comments (percentage of total comments) and total participants.

**Consolidated category**	**2016/2017**	**2017/2018**	**2018/2019**
Self-development as a researcher	14 (46.67%)	10 (37.04%)	13 (41.94%)
Collaborative process	11 (36.67)	6 (22.22%)	15 (48.39%)
HeNReG process	5 (16.67%)	11 (40.74%)	3 (9.68%)
**Total comments**	**30**	**27**	**31**
**Total participants**	**12**	**22**	**17**

### 3.2. Responses to HeNReG-related prompts

The responses HeNReG participants provided to the three prompts in 2017–2018 and the six prompts in the 2018–2019 (see Tables 3 and 4) that were specific to the HeNReG itself are examined here for the first time. They are relevant to those who may want to replicate this process because they indicate not only the satisfaction level of group members throughout the process (rather than just in relation to the year-end) they also demonstrate the process of question-asking from those questions that are most objective to those that are increasingly subjective. What follows is the responses given to the individual prompts of those group members who were present at the meeting when the prompt responses were discussed (not all members attended each session) as archived on the private Facebook group for each year. The responses themselves can be found in Table 8.

Regarding the response to the HeNReG-related “when” prompt, it can be seen in Table 8 that in asking this type of question participants for the most part focused on a specific time that they remember. There are a few instances in which the particular group member considers this question more abstractly—needing, reflecting, realizing or rediscovering. Nevertheless, the “when” prompt is inclined to invoke the most objective reply from participants.

What is interesting concerning the responses to the HeNReG-specific “where” prompt (see Table 8) is that, unlike the “when” prompt (which was seen to have the effect of potentially producing an abstract response) posing this prompt only resulted in objectively exact locations being provided in participant responses. This demonstrates that even though when something occurs is in principle more precise than where something takes place, because of the ability to use “when” to also mean “under what conditions”, the “when” prompt may produce a more subjective response if interpreted in this manner by the responder. This needs to be recognized by facilitators intending to make use of the process.

As can be the case with the prompts that have been presented so far, participants are able to use the prompt to answer what truly concerns them, rather than providing a direct response to the question. Referring to Table 8, one participant used the “who” prompt to inform other participants that this research method should be published—intimating that these future readers are the “who” the participant will inform about the HeNReG. For those group members who answered the question directly, it is interesting to learn that most had told others about the group and those who have been told were generally close to the participants. What wasn't determined is what participants told others about the group and whether there might have been a breach of confidentiality [94]. This possibility is something to consider and note in advance for those who are interested in starting a HeNReG-like group.

It can be recognized that with the “what” prompt seen in Table 8, the type of questioning is a balance of objective and subjective. If they found anything lacking in the HeNReG (three of them did not) the participants considered the question broadly in their response but also provide an example (although vague) of what might be done.

Respondents interpreted the first how prompt in two different ways as seen in Table 8. One was “what was the process that led to you being aware of the HeNReG as a group you might join”. The other was “how did you come to know of how the HeNReG might be of help to you”. Most answered regarding the first way; the last three responded with the second way as the focus. It is interesting to observe that of those who answered with respect to the first way all but one came to know of the group through personal encounters, either directly with the facilitator or with someone who might have personally experienced the group. For those interested in starting a similar group, the importance of networking in finding group members should therefore be noted.

In this second “how” prompt regarding the HeNReG (see Table 8), some of the participants, although they had been prompted to address how the HeNReG was lacking, chose instead to clarify the helpfulness of the HeNReG process. Others offered ways to improve the HeNReG: using Facebook more effectively, having more medical professionals involved, and encouraging the participants to be more attuned to the group process by being self-selective about what and how they contribute. These are things that those wanting to start their own HeNReG-type group might want to consider as a focus in improving upon the process.

The “how” prompt related to doodling found in Table 8 provided information on the way doodling affected the participants’ research related to health. Most group members were more inclined to answer with respect to their enjoyment of doodling, rather than how it may have affected their research. Things that were pointed out that could be interpreted as aiding their research are that doodling gave them a window into their thinking, reduced stress, encouraged additional creativity, and enhanced reflection. It should be noted, however, of those who did doodle, one felt “a bit of chaos” because of the lack of objectivity to doodles. As well, the participant acknowledged a fear of drawing. This type of fear holds for creativity in general when a participant is found lacking in mindfulness [95] and should be taken into consideration by those intending to encourage doodling in such a group.

Most participants denied that the HeNReG produced a feeling of being anxious or overwhelmed, as can be seen in the responses to the first “why” prompt in Table 8. For those who did feel additionally burdened in any way as a result of the HeNReG, it was because of the processes producing a self-encounter that showed them to be personally lacking in their own estimations. For these participants who remained self-critical even after engaging in most of this process (this “why” prompt was provided in the third to last week of meetings (see Table 4)), it is likely that the HeNReG process was insufficient to deal with their stress leading to their research-related burnout. Nevertheless, one of the participants who became more self-critical merely considered this “a brave new world”. Whether this was to be feared or embraced was not mentioned. Still, those initiating such a group intended to reduce burnout in participants should be aware that this group process may not reduce burnout entirely in some participants and that this type of “why” question may identify those participants who still feel burned out once the process ends.

The final HeNReG-related prompt found in Table 8, one provided as the last of the 2018–2019 year, was the most subjective question posed and made each participant truly think about what the HeNReG provided to them with respect to confronting their stress-induced burnout. In each case, the participants were able to identify at least one way in which the HeNReG enabled them to value their work. This is significant because all group members joined because, at that time, they no longer were able to find sufficient value to their research work because of experiencing burnout.

**Table 8.** Reponses of group members to writing prompts specific to information gathered regarding the HeNReG for the years 2016–2019 in the yearly meetings.

**Writing prompt**	**Responses to writing prompt**
When did you think the HeNReG could be of help to you?	
	1. When I have gathered enough “qualitative experience” to work with.
	2. When I first heard about the program
	3. It's hard to remember when I first thought this.
	4. As soon as I returned to the group.
	5. Realizing there is a limit on how far I can get in researching entirely on my own.
	6. HeNReG is very helpful to me when I need perspectives.
	7. When one of the medical participants spoke about self-esteem as a thing that has to be built and, when built right, can be used to weather any storm.
	8. When it made me reflect on my insights, perspectives, and thought processes.
	9. As soon as I figured out what the HeNReG does I knew it would be a good place for me to articulate my ideas in a safe and supportive environment.
	10. When I wanted to bring a secondary school program that I initiated to a higher education setting.
	11. My participation in the program was with the intent to ground myself again, and to rediscover why I entered the nursing profession in the first place.
Where were you when you first thought you needed the HeNReG to help with your research related to health?	
	1. I was spacing out when I thought I needed help with my research.
	2. I was still in school, doing what a student does, reconfiguring myself.
	3. When I was at a student government meeting and found that not only were my ideas not received as I expected, they created a result I didn't want.
	4. I was in the cafeteria of my graduate school program.
	5. I was starting my winter semester in 2^nd^ year and realized I was unmotivated to do my research and wasn't sleeping
	6. I was at home, working on my first publication.
	7. I was in my dorm, staring out the window when I thought I could use some help with my research.
	8. I was living in Sao Paulo, Brazil, working in a university lab.
	9. After I enrolled in the Master of Public Health program and I decided I needed a practical component and chose Occupational and Environmental Health.
	10. I never thought I needed help with my research until I joined the HeNReG
	11. I was in my office trying to rationalize why I couldn't proceed with my project that had been put on hold.
Who have you told about the research practice used at the HeNReG?	
	1. A few of my friends.
	2. I have read many books on how to improve research practice. This is the first method that uses questions from the most objective to most subjective. An article needs to be written about this research method to tell people.
	3. My parents. I always share interesting ideas with them.
	4. No one, but I probably should
	5. My family and my therapist
	6. My husband and my parents
	7. One of my peers I have known for 10 years who went to therapy to overcome anxiety
	8. I haven't discussed it with anyone
	9. A close friend who works in a sleep research lab
	10. I've told two people who I've been growing a friendship with for the past two years
	11. A friend from college and my parents
What would improve the usefulness of the HeNReG to your research related to health?	
	1. Extension of the focus of the individual narrative into the narratives of others.
	2. If the group didn't meet during working hours
	3. The group meets with all the things I consider important.
	4. The group is already useful in gathering different people from different backgrounds and trying to understand each other's narratives
	5. It's difficult to say, since my focus in health has shifted over this year.
	6. I'm not sure what would make this group more useful
	7. It would be great if other organizations used this method for professional development. I think it would be helpful for clinicians
	8. If the questioners focused on their perspective as employees to help me broaden my view of my workplace
How did you become aware of the HeNReG?	
	1. While working on my dissertation, I looked for narrative working groups... Interestingly, the HeNReG came up near the top of the search.
	2. I participate in the University of Toronto Hub on the Ten Thousand Coffees website and so does the facilitator.
	3. My aunt told me about it.
	4. I was in contact with the facilitator. She introduced this group to me and before this I didn't even realize I had a research question in mind.
	5. I first heard of this group in a discussion at home.
	6. I was vaguely aware of narrative therapy as a practice because that is the form of therapy that my therapist practises...I met the facilitator at a mentorship event.
	7. I met the facilitator at a college event.
	8. Through networking in my research related to health.
	9. I became aware of the HeNReG most recently as I was writing a chapter for a book on self-directed learning
	10. I became aware of the group as the weeks progressed in being part of it
	11. I'm self-aware with respect to most components, except one—that's where the Health Narratives Research Group comes in.
How could the HeNReG be of more help to you regarding your research related to health?	
	1. The HeNReG is already quite helpful to me when it comes to reflecting on my ideas and the progress made so far.
	2. I think it has been very helpful, to be honest.
	3. One way is to go deeper on Facebook.
	4. I think this is the perfect environment for me.
	5. From where I am now, I see the most useful changes happening naturally over time, as they have in the past, in relation to this group
	6. I would greatly appreciate the opportunity to meet with some members for informational interviews...and explore the possibility of collaboration in support of ongoing projects.
	7. I cannot think of any ways that wouldn't be an imposition.
	8. It could be of greater help if more medical professionals participated in the group.
	9. If we were all able to become more self-selective and expressive as a shared goal.
	10. If there were more participants—the questions are great!
	11. If the HeNReG were an open forum for discussion and we could go on learning from the members
	12. If we could go on a retreat together.
How has encouraging doodling at the HeNReG meetings affected your research related to health?	
	1. It has given me glimpses and starting points into what is in my subconscious and unconscious
	2. I don't get around to drawing in these sessions.
	3. It turns the space into a much freer and more relaxed space, intellectually and emotionally for me.
	4. The drawing exercises were useful for stress relief and great for clearing my head, but I'm not sure how it has affected my work.
	5. Doodling used to be very helpful for me. Now, it has given me a bit of chaos because there is no objective to these pictures. I guess I'm just afraid to draw now.
	6. Not sure if there are directly measurable effects; however, the doodling is certainly an additional creative outlet
	7. I'm a big proponent of working on things with your hands. I think making things, for example doodling, allows us to remember our body is more than just a car that carries our head from room to room.
	8. It makes the atmosphere more relaxing and friendly. It also helps me think creatively and in colours (literally too!).
	9. It gave me a lot of opportunity to reflect on the questions that were posed.
	10. It helped to reduce people's anxiety and offered a new aspect to the HeNReG.
	11. My original goal in joining the group was to introduce drawing to be part of the activities and encourage people to draw.
Why has the HeNReG contributed to you feeling anxious or overwhelmed?	
	1. It's a stretch to say that the HeNReG made me feel anxious.
	2. It has helped me feel calmer.
	3. It has made me aware of my lack of understanding.
	4. It has not contributed to me feeling anxious or overwhelmed in any way.
	5. It was a challenge for me to think of how anything I do comes together to form a story of my research goals.
	6. I feel a bit uneasy and anxious about sharing more.
	7. I've never been great at speed writing but the HeNReG as allowed me to expand upon my critical thinking and writing capabilities.
	8. To be honest, I don't think it did.
	9. Because of how impossible improvement feels.
	10. I always forget to do the online part.
	11. As a non-native English speaker, every opportunity of handwriting is very welcomed.
	12. When you lose that objective knowledge, it's stressful and overwhelming but it is better for your brain.
	13. It challenges me. It makes me self-conscious, self-critical. It stirs up cognitive dissonance. It makes me question myself. It throws me into a brave new world.
Why has the HeNReG helped you understand why you value your work?	
	1. The HeNReG is structured in such a way that I have been able to develop what it is I value about my work from what is most objective to what is most subjective.
	2. It's made me reflect a lot on what I'm currently doing and what I want to achieve in the near future. It also made me realize how much work I have ahead of me!
	3. In repeating something again and again, I find out if I really believe in it. I understand that I value my work when it is self-directed and I get to put my mark on it.
	4. It reminds me of the work that takes place during the research process, especially of the object that involves diverse discussion, so that fresh ideas can be sculpted by many.
	5. Because people here are so receptive and creative, it has helped me significantly in finding intrinsic value where there may not have been extrinsic.
	6. Knowing that I need to be prepared to answer questions about my research each week keeps it top of mind and prevents it being back burnered. The questions also helped me clarify my thinking and my opinions about my research.
	7. The HeNReG gives me the opportunity to think over my work since the prompts always make me ponder in a deeper way about my work and my role in society.
	8. I was able to reflect more on what my work entailed and find importance in all kinds of different aspects of it.
	9. The HeNReG has helped me understand the value of my work because I'm able to discuss it with people.
	10. The HeNReG provides a platform to help you reflect on what really matters to you.

## 4. Discussion

This discussion is divided into three parts. The first concerns comparing interventions that focus on developing resilience with the HeNReG for decreasing burnout. Second to be discussed is modifying the HeNReG from a research group to one that is appropriate for practitioners. Third, what this study adds will be presented.

### 4.1. Resilience interventions compared with the HeNReG

The HeNReG has been presented as a narrative-supporting group that has the potential to help participants improve their self-direction while decreasing burnout. It compares favorably with other interventions [41]–[43] that have been designed to decrease burnout in nurse practitioners as is evident in Table 9. Those studies compared are particular to public health practitioners that focused on reducing burnout in contrast to improving resilience, as improved resilience itself may not translate to burnout reduction [52].

**Table 9.** Interventions to decrease burnout in a health setting and their citation number in relation to decreased burnout, and improved self-direction.

**Intervention**	**Burnout found decreased**	**Self-direction improved**
THRIVE© program [41]	✓	✗
MSCR intervention [41]	✗	✗
MSC training [41]	✓	✗
CRM training [41]	✗	✗
SMART [41]	✓	✗
TM [42]	✓	✗
Mindfulness [43]	✓	✗
HeNReG [66]–[70]	✓	✓

Seven resilience-developing programs are offered for comparison with the HeNReG. These are discussed in three separate references [41]–[43]. Five of these come from the reference associated with one citation [41] as this reference is a review of literature on resilience interventions in nurses. Regarding this reference [41], in total, there are 15 studies included for review. Only 5 are considered here because the others do not mention the effect of the intervention on burnout or do not relate to public health nurse practitioners. Acronyms with which the interventions are known are listed in Table 9. The full names of these interventions are as follows: THRIVE© = Tool for Health and Resilience in Vulnerable Environments [96]; MSCR = mindfulness self-care and resiliency [41]; MSC = Mindfulness self-care [41]; CRM = Community Resiliency Model [41]; SMART = Stress management and Resilience training [41]; and TM = Transcendental Meditation [42].

In relation to each of these programs for improving resilience in nurses, the two to be noted initially are the programs in which there was no benefit to the two variables as a result of the intervention. Both of these studies are reported in [41]. For the MSCR intervention, the study found no significant different as a result of the intervention. With regards to the CRM training, although there were improvements in other areas as a result of the intervention, there were none for burnout reduction or self-direction. For two of the other studies—those of SMART [41] and of TM [42]—an improvement in burnout reduction is noted; however, this was not accompanied with a reporting of greater self-direction. In the other three resilience interventions, THRIVE© [41], MSC [41], and Mindfulness [43], each recorded a reduction in burnout. However, none of the studies mentioned whether there was an improvement in self-direction. As can be recognized, only the HeNReG has been able to reduce burnout, and positively affect self-direction. In this way, it is found superior to resilience programs for reducing burnout.

### 4.2. From research group to practitioner group

A notable difference between those who were part of the in-person meetings of the HeNReG from 2016–2019 and the proposed group for public health nurse practitioners is that they considered themselves researchers rather than practitioners. Thus, the first concern is how this program could be modified to correspond with nurse practitioners in the public health setting.

An important ingredient of the in-person meetings of the HeNReG is that the meetings took place in a non-preferential location, outside the workplace of any participant. As such, it is important for nurse practitioners experiencing burnout to have a location to meet with other participants that is unrelated to their daily work activities as location itself can contribute to burnout [97]. In this way, the suggestion by one of the HeNReG participants in replying to the prompt, “How could the HeNReG be of more help to you regarding your research related to health?”, is relevant—“go on a retreat together.” Organizing a retreat of participants in what would be a Health Narratives Practitioners Group (HeNPraG) would involve conducting the group in a neutral location and providing concentrated time to complete the responses to prompts—time that might not be available in the public health delivery setting in any other way, particularly for newly graduated nurses [98]. Nevertheless, according to one recent nursing publication regarding burnout, “The concept of retreats is interesting and could be beneficial for participants; however, it is unrealistic to expect staff, teams to attend weekend retreats, especially if these are self-funded” [99]. As such, if a retreat is decided as the best alternative to reduce burnout in public health practitioners, this retreat should be timed appropriately and supported by the employing institution. Positive results of such institution-supported retreats for all staff—physicians (faculty and residents), nurses, and other staff—recently have been published [100].

If self-direction is to be enhanced, it is important that those participating in the proposed HeNPraG be self-identified, rather than employer-selected. As such, the facilitator's notice to invite participation to join a HeNPraG must be widely circulated throughout the supporting institution and open to all employees who think they might find it beneficial as diversity of participation is important to the group [71]. Furthermore, this facilitator of the group must be someone considered impartial and trustworthy by all potential participants as it has been noted that what leaders say and do makes up 70% of what participants report regarding feeling included [101]. One suggestion is that the hospital bioethicist, as the institutional representative representing these qualities [102], be encouraged to assume this position. The role would then be similar to that assumed by bioethicists during COVID-19 as resilience coaches [103]. The prompts that have been provided in Tables 3 and 4 can serve as examples of those that such a facilitator could provide to the group members, substituting “public health work” for “research related to health”. As well, the facilitator would do well to consider encouraging, and providing the materials for, doodling during the meeting period, as doodling has been shown especially effective in reducing the stress of nurse practitioners [104].

To evaluate the effectiveness of a potential HeNPraG, the facilitator of the group is advised to include writing prompts that relate to reflecting on the HeNPraG in each of the six different types of prompts (when, where, who, what, how, and why) as well as in the final evaluation of the program offered. Examples of the types of prompts that could be included to evaluate the HeNPraG are provided in Table 8, as well as the responses to these prompts that were provided by HeNReG participants when they were prompted. The questions that facilitators might include in the final evaluation of the program would appropriately modify those asked of HeNReG participants: “How was the group valuable to you in your public health work?” (Table 5), and “How might the HeNPraG be of help to you in the future?” (Table 6).

### 4.3. What this study adds

This study has advanced the literature by providing suggestions for how the HeNReG might be modified as a HeNPRaG to be an appropriate and effective group for self-direction development and burnout reduction in public health nurses. As well it has examined evidence that, unlike the HeNReG, resilience training alone is insufficient to increase self-direction while reducing burnout. This is the first study to undertake approaching burnout reduction in public health nurse practitioners in this regard and provides valuable information as burnout reduction and improved self-direction are both necessary for career sustainability in public health nurse practitioners [105].

## 5. Conclusions

Public health nurse practitioners as professionals are necessarily exposed to stress in their daily work activities to the extent that burnout is the result. Recent changes in the understanding of gender have served both to increase the diversity of those who choose to be nurses and add stress to the profession as a result of blurred professional lines between nurses and physicians. The 2020–2023 COVID-19 pandemic brought to the public's attention the importance of improving the mental health of public health providers that had diminished as a result of burnout. To reduce this burnout, the development of self-direction is necessary. One way found effective in researchers to accomplish this is through a method like that of the HeNReG. The results of the in-person meetings of that group between 2016–2019 have demonstrated that this type of narrative group can, in contrast to interventions to improve resilience, develop self-direction and reduce burnout from considering what is most objective regarding their relation to their work to what is most subjective. How this process, developed for researchers, can be modified for practitioners in public health has been presented as a Health Narratives Practitioner Group (HeNPraG) and considered likely to be most effective if conducted as a team retreat facilitated by the person performing the role of hospital bioethicist as an objective and trusted ally. The development and effectiveness of such narrative groups for reducing burnout in nurses requires and points to future research.

### 5.1. Limitations

A limitation is that this account did not discuss the potential of online-only participation in a HeNPraG if it were to be formed. Furthermore, the author specifically did not discuss the archived results of the HeNReG during the period when it was conducted entirely online as a result of COVID-19 limitations. Online participation was not discussed because it was found that once the HeNReG moved entirely online, although group members continued to participate with the facilitator in a manner comparable to pre-COVID-19, the group members did not choose to interact with each other. If the point of the proposed HeNPraG is to encourage collaborative self-direction among public health practitioners then online meetings have been found unlikely to be of help in promoting the necessary team mindfulness [69] to reduce burnout in nurses.

Another limitation is that this report is historical research rather than an empirical study that was approved by a research ethics board. This means that the report was not structured as an empirical study to include samples and data, measures of variables, models, and the data analysis procedure. On the other hand, if this were a research study, and participants knew it to be such, they may not have agreed to join the group or, if they did, they may have felt additional anxiety from participating in a study. Given that the point of the group was to act as an offering to reduce research burnout, rather than conduct research on the group, it may be best that it was not an empirical study as earlier research found that those with burnout are generally unwilling to participate in research studies [106].

A further limitation is that this study is unable to provide directives on how public health policy might be modified as a result of the recognized need to decrease burnout in nurse practitioners. As no review was conducted of current public health policies related to nurse practitioners, it would be overstepping the bounds of what was investigated to make suggestions regarding changes to public health policy. Nevertheless, that these potential changes weren't discussed is a limitation.

## Use of AI tools declaration

The author declares no Artificial Intelligence (AI) tools have been used in the creation of this article.


