# Supplementary material for: The Health Narratives Research Group (HeNReG): A self-direction process offered to help decrease burnout in public health nurse practitioners
Source: AIMS Public Health. 2024 Feb 6;11(1):176–208. doi: 10.3934/publichealth.2024009 (PMC11007417; doi:10.3934/publichealth.2024009)
Supplement: Supplementary file 1 [file publichealth-11-01-009-s001.pdf]

AIMS Public Health

AIMS Public Health, 11(1): 176–208.
DOI: 10.3934/publichealth.2024009
Received: 06 October 2023
Revised: 27 January 2024
Accepted: 30 January 2024
Published: 06 February 2024

http://www.aimspress.com/journal/aimsph

Research Article

The Health Narratives Research Group (HeNReG): A self-direction process offered to help decrease burnout in public health nurse practitioners

Carol Nash*

History of Medicine Program, Department of Psychiatry, Temerty Faculty of Medicine, University of Toronto, Toronto, ON M5S 1A1

Correspondence: Email: carol.nash@utoronto.ca.

Supplementary

The Health Narratives Research Group (HeNReG)

What it is:

-The HeNReG is a yearly, free, non-credit, university-wide, democratic group that has been offered in association with the Health, Arts and Humanities Program of the Department of Psychiatry in the University of Toronto since 2015.
-The facilitator/founder/originator is Carol Nash PhD, Scholar in Residence, History of Medicine Program, Department of Psychiatry, Faculty of Medicine, University of Toronto who participates as a equal members of the group.
-The group meets weekly on Wednesdays from 4–6 p.m. from the first week in October to the last week in April in room 941, Mount Sinai Hospital.

Philosophy of the HeNReG:

-Truth is considered to be like a landscape with obstacles.
-Each member is equal in having a position on the landscape.
-The type of obstacles each member has related to their position determines their point of view.
-To try to visualize the landscape is to re-search.
-To re-search is what is assumed by the word "research" for the purpose of the group.
-There are two ways to research in this analogy: discipline-based and narrative.
-In discipline-based research:
    -Obstacles in the landscape as barriers to eliminate through climbing higher.
    -Higher views supersede lower ones because research is considered hierarchical.
    -The purpose is to create the most accurate aerial view of the landscape.
    -The view at the top is the desired view.
-In narrative research:
    -Obstacles in the landscape as landmarks to find a route around.
    -Each person's point of view is equal
    -The routes created from one point of view to another can be added together.
    -All points of view are necessary in navigating the landscape.

-The HeNReG accepts the value of discipline-based research but uses narrative research to create as many routes as possible from one member's point of view to that of other members, similar to a "street view" in Google maps.

Joining:

-Potential members email the facilitator stating their interest in joining the HeNReG and indicating their research interest related to health.
-Membership requires a willingness to either attend the meetings in person or to participate online.
-Members can join at any time during the year.
-Regular attendance is not required.
-<u>By joining, members agree their work may be anonymously referenced in presentations given and/or scholarly articles written by the facilitator regarding the yearly results of the HeNReG.</u>

Returning members:

-In September, the previous year's members will be contacted by the facilitator and asked if they would like to continue as members of the HeNReG.
-Returning members who previously participated in person can decide to become online members and vice versa.
-<u>By rejoining, returning members agree their work may be anonymously referenced in presentations given and/or scholarly articles written by the facilitator regarding the yearly results of the HeNReG.</u>

At the start of the meetings:

-Members sit in a circle around the table.

-Members are provided with paper, pens and pencils but those who prefer to use their phones for writing are encouraged to do so.
-At the beginning of each meeting, the facilitator provides a prompt to write to, stream of consciousness.
-Depending on how many people are at the meeting, the response to the prompt is for 5 minutes of writing or less.
-The initial prompt for the year asks each person to describe themselves with respect to their research related to health.

The structured prompts and responses to them:

-The prompts that follow in the weeks to come ask group members to consider what is most known (or objective) with respect to their research related to health to what is most vague (or subjective).
-To facilitate this "objective to subjective" consideration of their research related to health, the questions over the year will be ordered as follows: when, where, who, what, how, why.
-There will be more than one session devoted to each type of question (usually four).
-Responses are to be stream of consciousness.
-Members who participate online will be given the prompt of the week the day before the meeting by the facilitator over Messenger so that they have time to reply to the prompt online before the meeting
-Once people have completed their written response to the weekly prompt, each person at the meeting reads their response, one by one.
-Each person is asked to read in relation to where they sit, going around the circle.

Question asking:

-After a person has read their response to the prompt, each member of the group is given the opportunity to ask one question of the person who has read to further clarify what has been read.
-When each person asks a question is with respect to where they sit, going around the circle.
-The questions people are to ask relate to the type of question posed for the prompt that week, i.e., if a "who" question is asked, each person asks a clarifying "who" question.
-If a group member doesn't have a question, that person can pass.
-If a group member can't think of a question right away they get another opportunity after going around the circle.
-People wait their turn to speak in the circle as members are asked not to put up their hands in order to reduce competition to speak and support the democratic nature of the group.
-When each question is asked, the person who has read their reply to the prompt answers the question asked of them.
-When each person has asked a question of the reader, if time permits, the person who has read is asked if they would like to give a summary of how these questions may have helped them clarify their research.
-Members should aim to make their questions and responses as short as possible so everyone has an equal chance to speak.
-Once everyone present at the meeting has provided their questions to each of the readers, the facilitator will read the responses to the prompt that have been provided by the online participants.
-Those at the meeting will then provide questions to each online participate going around the circle.

-The facilitator takes notes on all questions asked and replies given.
-At the end of the meeting, the responses to the prompts are collected by the facilitator to be posted—along with the questions and replies—to the yearly, private Facebook group.
-Once posted, online members no have the opportunity to reply online to the questions they have received.

Drawing:

-Everyone also has the opportunity to draw during the meeting.
-Members are provided with paper, and various art supplies.
-Drawing prompts at times will be given in addition to the writing prompts.
-When drawing prompts are not provided, members are encouraged to doodle.
-Drawing can be done throughout the meeting at any time members are not otherwise engaged with writing or posing questions to the person who has read their response to the writing prompt.
-At the end of the meeting, members are asked to describe their drawings one at a time going around the circle.
-The facilitator notes down all the descriptions of the drawings.
-Online members are encouraged to include drawings that they might do as well along with their description.
-At the end of the meeting, the drawings are collected by the facilitator and posted to the online Facebook group.

Extending the time of the group:

-If, to hear from everyone, the question-posing is likely to go over two hours, members will be asked ahead of time if they are willing to go over the two-hour time scheduled.
-There are some dates when staying overtime is not possible because the room is booked.
-If the meeting cannot be extended, to ensure that everyone can be equally heard, the time limit of the prompt will be reduced from 5 minutes.

Online participation:

-A secret Facebook group is set up yearly by the facilitator.
-All members of the group who are Facebook friends of the facilitator are invited to the Facebook group.
-The facilitator posts responses to the prompts and questions asked and answered for each member.
-The facilitator posts the drawings and their descriptions for each member.
-Members are encouraged to ask further questions to each other on the secret Facebook group
-Members who can’t make the meeting in person can participate by replying to the writing prompt given by the facilitator a day before the meeting via Messenger.
-Their replies will be read by the facilitator at the meeting and questions to the member will be noted by the facilitator who will post them to the private Facebook group after the meeting.
-Members are encouraged to read the posts on the private Facebook group and respond to questions that they receive from other members if they are not at the meeting.
-Members are also encouraged to post relevant links to the private Facebook group.

Feedback:

-Feedback for the HeNReG will be formally requested twice per year, at the last meeting in December and the last meeting in April.
-The feedback form will be one common to the Health, Arts and Humanities Program of the Department of Psychiatry.
-The feedback form will be a Google survey to be completed online.

AIMS Press

© 2024 the Author(s), licensee AIMS Press. This is an open access article distributed under the terms of the Creative Commons Attribution License (http://creativecommons.org/licenses/by/4.0)
